# Supplementary material for: Spatial distribution of complete basic childhood vaccination and associated factors among children aged 12–23 months in Ethiopia. A spatial and multilevel analysis
Source: PLoS One. 2023 Jan 6;18(1):e0279399. doi: 10.1371/journal.pone.0279399 (PMC9821487; doi:10.1371/journal.pone.0279399)
Supplement: Attachment — Submitted filename: Response to Reviewers.docx [file pone.0279399.s002.docx]

**Authors’ response to reviews**

**Manuscript title: Spatial distribution of complete basic childhood vaccination and associated factors among children aged 12-23 months in Ethiopia. a spatial and multilevel analysis.**

**Authors:**

Getanew Aschalew Tesfa ([getanewaschalew@gmail.com](mailto:getanewaschalew@gmail.com))

Delelegn Emwodew Yehualashet ([delelegn1244@gmail.com](mailto:delelegn1244@gmail.com))

Addisu Getnet ([addisuge@du.edu.et](mailto:addisuge@du.edu.et))

Binyam Tariku Seboka ([bini555tar@gmail.com](mailto:bini555tar@gmail.com))

**Version:** 1 **Date:** 20 May 2022

**Author’s response to reviews:**

Dear, Editors of PLOSONE

It has been recalled that we the authors of the manuscript entitled “Spatial distribution of complete basic childhood vaccination and associated factors among children aged 12-23 months in Ethiopia. a spatial and multilevel analysis.” submitted our manuscript for publication in your journal and received reviewers and editors comments for the betterment of the manuscript before its publication.

In line with this, all authors are very happy with the constructive and valuable comments given by both reviewers and the editor. Accordingly, we have considered all the comments and provided a point-by-point response and explanations for all the questions raised.

Finally, we have submitted all the required documents in their revised form. We hope that we have addressed all the questions and if you have any points for further clarity, let us know.

All the authors would like to thank the editorial team

S.no	Academic editor /Reviewer comments	Authors’ Responses

Academic editor comments:

1. Please ensure that your manuscript meets PLOS ONE's style requirements, including those for file naming. Thank you for the comment. We tried to revise the manuscript based on PLOS ONE's style requirements.
2. Please provide additional details regarding participant consent in the Methods section. Thank you for the comment. We explained it. Refer page 7 line 171-175.
3. Please note that PLOS ONE is unable to publish previously copyrighted maps or satellite images, or images created using proprietary data. For these reasons, we cannot publish images generated by software which copyrights their output (such as Google Maps, Street View, and Earth). In order to use these images in your submission, we require explicit permission from the copyright owner to publish the figures under the CC BY 4.0 license.

At this time, please kindly clarify the following regarding Figure 2:

a) Where did the authors obtain the maps, basemaps, shapefiles, map data, etc. in Figure 2?
b) If any of the map images have been previously copyrighted, we require specific consent from the copyright holder to publish these images in PLOS ONE, under the CC BY 4.0 license.

**Authors' response:** Editor, thank you for the concern. Figure 2 is not copyrighted rather we have done using ArcGIS and SaTScan software based on the shapefile of Ethiopia received from Ethiopian Central Statistical Agency (CSA) and GPS data (longitude and latitude) from the measure demographic and health survey (DHS) program by explaining the objective of the study through online requesting and allowed us to access the shapefile and GPS data. Because we need to analyze the spatial distribution of complete basic childhood vaccination, we cited the shapefile's source. Therefore, the map presented in our study is not copyrighted; rather it shows the result of our spatial analysis.

c) Were any sample data used in the map in Figure 2 proprietary data (e,g,. from ArcGIS and/or SaTScan)?

**Authors’ response:** Thank you for the concern, editor. We used ArcGIS and SaTScan statistical software to illustrate the spatial distribution of complete basic childhood vaccination using data from the measure DHS program. After describing the objective of using the data, we obtained the data and acquired approval from the Measure DHS program. The DHS dataset is open to the public, and figures obtained as a final analysis result are not copyrighted.

4. Please review your reference list to ensure that it is complete and correct. Thank you. We tried to review it carefully.

Reviewer #1:

1. I suggest to edit the text and to slightly modify the abstract, specifying that some of those factors are associated with complete vaccination (i.e. mother age or religion) while other with incomplete vaccination (i.e. some specific regions). Thank you very much for the comments. We tried to revise it carefully.

Reviewer #2:

1. The manuscript contains numerous typographical errors that should be addressed in the revised manuscript. Thank you very much for the comment. We tried to make a substantial revision on the typography.

2. You conducted a four-model multilevel binary logistic regression analysis. My main concerns are:

- Have you checked the interaction between individual level factors with the level factors? For instance, interaction between religion and regions of the participants?
- If no why? and If so, what were your findings? Thank you for the comment. Refer page 6 line 156-159.

3. You've used a null model (a model without any variables). What was the percentage of complete basic childhood vaccination that could be explained by level 2 factors? To what extent does one's place of residence and/or region explain complete basic childhood vaccination? What exactly was the ICC? Was it significant? Thank you for your interesting comment. We included it based on your comment. Refer page 13 line 253-259 and Table 4 page 16.
4. The manner in which you write the relationship between the individual level factor age and complete vaccination is not as simple. Please keep it short and simple for the layperson to understand. Thank you for the comment. We rephrased it. Refer page 13 line 270-273.
5. How can inequalities in immunization service be used to justify low coverage of complete basic childhood vaccination? Do you mean that Ethiopia's immunization coverage is low? If this is the case, could you please provide evidence and explain how immunization coverage affects complete basic childhood immunization? Thank you for the comment. We corrected it. Refer page 17 line 306-311.
