# Supplementary material for: Spatial distribution of complete basic childhood vaccination and associated factors among children aged 12–23 months in Ethiopia. A spatial and multilevel analysis
Source: PLoS One. 2023 Jan 6;18(1):e0279399. doi: 10.1371/journal.pone.0279399 (PMC9821487; doi:10.1371/journal.pone.0279399)
Supplement: Attachment — Submitted filename: Response to Reviewers_v2.docx [file pone.0279399.s003.docx]

**Authors’ response to reviews**

**Manuscript title: Spatial distribution of complete basic childhood vaccination and associated factors among children aged 12-23 months in Ethiopia. a spatial and multilevel analysis.**

**Authors:**

Getanew Aschalew Tesfa ([getanewaschalew@gmail.com](mailto:getanewaschalew@gmail.com))

Delelegn Emwodew Yehualashet ([delelegn1244@gmail.com](mailto:delelegn1244@gmail.com))

Addisu Getnet ([addisuge@du.edu.et](mailto:addisuge@du.edu.et))

Kirubel Biweta Bimer ([kirubelbimer@gmail.com](mailto:kirubelbimer@gmail.com))

Binyam Tariku Seboka ([bini555tar@gmail.com](mailto:bini555tar@gmail.com))

**Version:** 2; Date**:** 7 November 2022

**Author’s response to reviews:**

Respected editor and reviewer, I tried to incorporate all comments in point-by-point response in the document as follows.

General comment (Journal Requirements)

1. Please review your reference list to ensure that it is complete and correct. If you have cited papers that have been retracted, please include the rationale for doing so in the manuscript text, or remove these references and replace them with relevant current references.

**Response:** thank you very much for your suggestion. We tried to revise our reference and change was made on the following references.

- L Arevshatiana, C.C., SK Lwangac, AO Misored, P Ndumbee, JF Sewardf, P Taylorg. *An evaluation of infant immunization in Africa: is a transformation in progress?* ; Available from: <https://www.who.int/bulletin/volumes/85/6/06-031526/en/>.: **Removed and** **Replaced by:** Lindstrand, A., et al., *The World of Immunization: Achievements, Challenges, and Strategic Vision for the Next Decade.* The Journal of Infectious Diseases, 2021. **224**(Supplement_4): p. S452-S467.
- Organization, W.H. *Immunization, Vaccines and Biologicals*. Available from: <https://www.who.int/immunization/documents/immunological_basis_series/en/>. : **removed**
- Ministry of Health-Ethiopia. *Expanded Program on Immunization (EPI)*. Available from: <https://www.moh.gov.et/ejcc/am/EPI>. **Removed and** **replaced by:** Belete, H., et al., *Routine immunization in Ethiopia.* The Ethiopian Journal of Health Development, 2015. **29**.
- 14.	Federal Ministry of Health, E. *ETHIOPIA NATIONAL EXPANDED PROGRAMME ON IMMUNIZATION COMPREHENSIVE MULTI-YEAR PLAN 2011 - 2015*. 2010; Available from: [https://bidinitiative.org/wpcontent/files_mf/1405630243EthiopiaComprehensivemultiyearplanfor20112015Year2010.pdf](https://bidinitiative.org/wp-content/files_mf/1405630243EthiopiaComprehensivemultiyearplanfor20112015Year2010.pdf). **Removed and** r**eplaced by:** Boulton, M.L., et al., *Vaccination timeliness among newborns and infants in Ethiopia.* PLOS ONE, 2019. **14**(2): p. e0212408.

Editor comment

2. Please, address previous reviewer comments properly. Why you not calculated the PCV (proportion of change in variance for Model I, II, II), and better to calculate the median odds ratio for all models as well?

**Response:** Thank you for your insight. We have already addressed it. Please refer page 14, line 280-290 and Table 4, page 16.
