# Supplementary material for: Spatial distribution of complete basic childhood vaccination and associated factors among children aged 12–23 months in Ethiopia. A spatial and multilevel analysis
Source: PLoS One. 2023 Jan 6;18(1):e0279399. doi: 10.1371/journal.pone.0279399 (PMC9821487; doi:10.1371/journal.pone.0279399)
Supplement: Attachment — Submitted filename: Response to Reviewers_v3.docx [file pone.0279399.s004.docx]

**Authors’ response to reviews**

**Manuscript title: Spatial distribution of complete basic childhood vaccination and associated factors among children aged 12-23 months in Ethiopia. a spatial and multilevel analysis.**

**Authors:**

Getanew Aschalew Tesfa ([getanewaschalew@gmail.com](mailto:getanewaschalew@gmail.com))

Delelegn Emwodew Yehualashet ([delelegn1244@gmail.com](mailto:delelegn1244@gmail.com))

Addisu Getnet ([addisuge@du.edu.et](mailto:addisuge@du.edu.et))

Kirubel Biweta Bimer ([kirubelbimer@gmail.com](mailto:kirubelbimer@gmail.com))

Binyam Tariku Seboka ([bini555tar@gmail.com](mailto:bini555tar@gmail.com))

**Version:** 3; Date**:** 15 November 2022

**Author’s response to reviews:**

Respected editor and reviewers, thank you for your valuable comments and suggestions. We made an effort to address every comment in the following point-by-point response.

1. Have you checked the interaction between individual level factors with the level factors? For instance, interaction between religion and regions of the participants? If no why? and If so, what were your findings?

**Response:** Thank you for your comment. Understanding geographical distributions of complete basic childhood vaccination and identifying associated predictors is crucial for designing effective policies and strategies that help to improve vaccination coverage. Keep in mind this, the first main specific objective of our study was to identify the spatial distribution of complete basic childhood vaccination. To attain this objective and to answer the question of where the hotspots for low vaccinations are located, spatial statistical analysis is required. So, our spatial analysis looked at only the dependent variable. Second, to identify potential predictor variables, regression analysis is required. Hence, we used multilevel multivariable logistic regression analysis. As stated above we identified predictors using regression analysis and regression models consider linear relationships (stationary relationships). Since one of the assumptions of regression models is that relationships are constant across the entire study area. Therefore, estimates generated in this model assume averages. Due to that, we didn't consider the interaction between individual-level factors with the level factors. However, we have checked the absence of multicollinearity between these independent variables using the variance inflation factor.

2. The manner in which you write the relationship between the individual level factor age and complete vaccination is not as simple. Please keep it short and simple for the layperson to understand.

**Response:** we revised it accordingly and corrected it as follows

“Maternal age was significantly associated with complete basic childhood vaccination. Children born to mothers who were 20-24 years old were approximately two times (AOR=2.12, 9S%CI: 1.06-4.24) more likely to be fully vaccinated than children whose mothers were 15-19 years old.” Refer page 13, line 263-266 (on the tracked version)

3. How can inequalities in immunization service be used to justify low coverage of complete basic childhood vaccination? Do you mean that Ethiopia's immunization coverage is low? If this is the case, could you please provide evidence and explain how immunization coverage affects complete basic childhood immunization?

**Response:** Thank you for your constructive comment. We mean that variability of immunization service access across areas might be one of the reasons for the low prevalence of complete basic childhood vaccination. Since our study was done based on the national representative data that took into account both rural and urban areas, the overall prevalence of complete basic childhood vaccination was varied compared to previous studies that were done in specific districts. Anyways, in order to minimize such type of misunderstanding, we have made some modifications in that sentence and additional explanations were added in the main document. Refer page 17, line 305-309 (on the tracked version)
