# Supplementary material for: Spatial distribution of complete basic childhood vaccination and associated factors among children aged 12–23 months in Ethiopia. A spatial and multilevel analysis
Source: PLoS One. 2023 Jan 6;18(1):e0279399. doi: 10.1371/journal.pone.0279399 (PMC9821487; doi:10.1371/journal.pone.0279399)
Supplement: Attachment — Submitted filename: Reviewer comments.docx [file pone.0279399.s001.docx]

**Manuscript title: Spatial distribution of complete basic childhood vaccination and associated factors among children aged 12-23 months in Ethiopia. a spatial and multilevel analysis.**

**Reviewer comments:**

The authors presented an intriguing public health issue in Ethiopia. I thoroughly read the manuscript and well written. However, I have some questions for the authors that I would like to see included in the revised manuscript.

1. The manuscript contains numerous typographical errors that should be addressed in the revised manuscript.

2. You conducted a four-model multilevel binary logistic regression analysis. My main concerns are:

- Have you checked the interaction between individual level factors with the level factors? For instance, interaction between religion and regions of the participants?
- If no why? and If so, what were your findings?

3. You've used a null model (a model without any variables). What was the percentage of complete basic childhood vaccination that could be explained by level 2 factors? To what extent does one's place of residence and/or region explain complete basic childhood vaccination? What exactly was the ICC? Was it significant?
4. The manner in which you write the relationship between the individual level factor age and complete vaccination is not as simple. Please keep it short and simple for the layperson to understand.
5. How can inequalities in immunization service be used to justify low coverage of complete basic childhood vaccination? Do you mean that Ethiopia's immunization coverage is low? If this is the case, could you please provide evidence and explain how immunization coverage affects complete basic childhood immunization?
